# A Study of Maternal Anemia and Utilization of Antenatal and Postnatal Care Services in Devbhumi Dwarka, Gujarat

**DOI:** 10.7759/cureus.30427

**Published:** 2022-10-18

**Authors:** Somen Saha, Apurva Kumar Pandya, Devang Raval, Mayur B Wanjari, Deepak Saxena

**Affiliations:** 1 Public Health, Indian Institute of Public Health Gandhinagar, Gandhinagar, IND; 2 Public Health, Parul Institute of Public Health, Vadodara, IND; 3 Research, Jawaharlal Nehru Medical College, Datta Meghe Institute of Medical Sciences (Deemed to be University), Wardha, IND; 4 Public Health, School of Epidemiology and Public Health, Jawaharlal Nehru Medical College, Datta Meghe Institute of Medical Sciences (Deemed to be University), Wardha, IND

**Keywords:** india, gujarat, devbhumi dwarka, post natal care, antenatal care, maternal anemia

## Abstract

Background and objective

Despite significant gains and achieving progress in the last decade, maternal anemia remains a major public health concern in India. Both antenatal (AN) and postnatal (PN) women are populations adversely affected by anemia. Reducing anemia among AN and PN women is one of the national priorities of Anemia Mukt Bharat. The present study aimed at assessing the prevalence of anemia, utilization of AN and PN care (ANC and PNC) services, and drivers of anemia among pregnant and lactating women (PLWs) in Devbhumi Dwarka district, Gujarat.

Methods

A descriptive cross-sectional study was conducted in four blocks of Devbhumi Dwarka district, Gujarat. A total of 1,185 PLWs were interviewed. Anemia was determined based on the last Hb test record on the Mamta Card (Mother and Child Protection Card).

Results

The mean age of the study population was 25.19 ±3.91 years: 24.95 ±3.94 years for pregnant women and 25.45 ±4.01 years for lactating women. The prevalence of anemia among pregnant women (Hb: <11 g/dL) was 72.92%; 33.91% had moderate and 0.83% had severe anemia. The prevalence of anemia among lactating women (Hb: <12 g/dL) was 26%; 63.93% had moderate and 1.29% had severe anemia. The utilization of nutrition and health services was found to be limited. Of the other sociodemographic factors, age (p=0.045), birth spacing (p=0.014), and education (p=0.017) were significant determinants of anemia among pregnant women, whereas parity (p=0.002), birth spacing (p=0.003), religion (p=0.041), and receipt of take-home ration (THR) (p=0.018) were significantly associated with anemia among lactating women.

Conclusion

The study revealed a high prevalence of anemia among PLWs and sub-optimal utilization of nutritional and healthcare services in Devbhumi Dwarka. Implementing interventions such as comprehensive nutrition education and counseling can contribute toward improving maternal and child health outcomes.

## Introduction

Maternal anemia adversely impacts maternal, neonatal, and child health outcomes [1]. Moreover, anemia and its related conditions have an intergenerational effect, characterized by repeating cycles of malnutrition and poverty in the long run [2]. According to the National Family Health Survey 4 (NFHS-4), the prevalence of anemia among antenatal (AN) women was 50.3%, while it was 57.9% for postnatal (PN) women [3]. India is home to the largest number of anemic pregnant women, accounting for about 80% of maternal deaths caused by anemia in South East Asia [4,5].

Maternal anemia is considered a risk factor for poor pregnancy outcomes. Studies have established that pregnant women with anemia are at increased risk of having low-birthweight (LBW) infants [6-9]. LBW is associated with poor health and nutritional outcomes later in life, such as poor growth and development, as well as increased morbidity and mortality in children [10]. Furthermore, severe iron-deficiency anemia has been associated with preterm births [11-12], poor anthropometric measures [13,14], fetal neural development [15], birth asphyxia [16], and increased maternal mortality [17]. 

Although studies on the impact of anemia during lactation are scarce, available evidence suggests a possible association of anemia among lactating women. Anemia among lactating women is associated with decreased immunity, diminished quality or volume of breast milk, increased susceptibility to infections, child inflammation [18], and delayed developmental outcomes in children [19]. It has also been associated with cognitive impairment, impaired quality of life, as well as postpartum depression [20]. These devastating impacts make anemia in lactating women one of the priority areas in global healthcare, especially in developing countries [20]. However, field observations by authors indicate a limited focus on anemia in lactating women compared to AN women by frontline health workers.

Both AN and PN care (ANC and PNC) play a crucial role in improving overall maternal nutrition and health status. Increased access to skilled care focusing on “1000-days”, from the critical pre-pregnancy period till children reach two years of age, has been recognized as a crucial factor contributing to improved maternal and child health and nutrition outcomes [20,21]. However, scaling up access to these interventions and increasing their utilization poses a huge challenge [22,23]. Various studies [24-26] have reported sub-optimal utilization of ANC and PNC services.

The World Health Organization (WHO) has set a global target of a 50% reduction of anemia among women of reproductive age by 2025 [27] and the Anemia Mukt Bharat initiative aims to reduce anemia among pregnant women to 32% and that among lactating women to 39.7% by 2022 [28]. To achieve this target, tackling anemia during pregnancy and the lactation period should be accorded great importance. 

A comprehensive understanding of the status of anemia among pregnant and lactating women (PLWs), ANC and PNC utilization, and factors associated with anemia among the population of Devbhumi Dwarka district, Gujarat is lacking. Hence, we conducted a cross-sectional survey of PLWs with the objectives to assess the status of anemia, utilization of ANC/PNC services, and examine the factors that cause anemia in Devbhumi Dwarka.

## Materials and methods

A descriptive cross-sectional design was adopted for this study. The study was conducted from February to March 2020 in four blocks of Devbhumi Dwarka district, Gujarat: Khambhaliya, Bhanvad, Kalyanpur, and Dwarka.

The sample size for this population-based survey was calculated using the OpenEpi program and assuming a 20% non-response rate. The sample size for the study was 1,185. To calculate the sample size, a population size of 130,000 for AN and PN women was assumed. The calculated sample size was 1,200 with a relative allowable error of 15% and design effect of 1 at a 95% significance level (alpha risk of 5%) by using the following formula: N=Z p (1-p)/D [Where p is prevalence and D is absolute precision (Z=1.96)].

We employed simple random sampling wherein samples were chosen by chance to elude sampling bias. A list of AN and PN women was obtained from the mHealth application (known as TeCHO+) managed by the government. All participants were de-identified and assigned a unique ID. Computer-generated random numbers were assigned to each odd-numbered participant. When a participant was not available in the field, we considered the next odd-numbered participant from the list for inclusion in the study. Operational definitions of various terms are presented in Table 1.

**Table 1:** Operational definitions

Term	Operational definition
Antenatal (AN) women	Women in the period of pregnancy till delivery, comprising 40 weeks. divided into the first, second, and third trimesters of pregnancy
Postnatal (PN) women	Women in the period from delivery till six weeks after delivery
Antenatal care (ANC) [29]	Care provided during the period of pregnancy till delivery (40 weeks)
Postnatal care (PNC) [30]	Care provided during the period from delivery till six weeks after delivery

Anemia was determined based on the last Hb test record on the Mamta Card (Mother and Child Protection Card). The anemia status of the surveyed women was classified into severe, moderate, mild, and any anemia based on WHO guidelines [31]. As per Intensified National Iron Plus Initiative (I-NIPI) guidelines [32], severe anemia level was defined as Hb <7.0 g/dL for AN women, and <8.0 g/dL for PN women. Moderate anemia was defined as a Hb level of 7.0-9.9 g/dL for AN women, and 8.0-10.9 g/dL for PN women. Mild anemia was defined as a Hb level of 10.0-10.9 g/dL in AN women, and 11.0-11.9 g/dL in PN women. Any anemia was defined as a Hb level of <11.0 g/dL in AN women, and <12.0 g/dL in PN women.

We considered socioeconomic and demographic factors such as age, gravidity, religion, caste, education, utilization of nutrition and services, and hygiene and sanitation practices as independent variables for the chi-square analysis. Descriptive statistics such as frequency, mean, and percentage were computed to describe the characteristics of the sample. A chi-square analysis was carried out, and an association between independent and dependent variables was measured. A p-value <0.05 was considered statistically significant. Necessary approval from the District authority was sought for conducting baseline analyses in the district. Written consent was sought from each study participant, and formal approval was obtained from state and district authorities. The Institutional Ethics Committee of the Indian Institute of Public Health Gandhinagar issued the IRB approval.

## Results

The mean age of AN and PN women was 25 ±3.91 years. About 62.92% of AN women and 58.01% of PN women were in the age group of 18-25 years. Among AN women, the mean age at marriage was 20.04 ±2.69 years, and that for PN women was 19.9 ±2.51 years. About 30.80% of AN and PN women had third or more gravida status whereas nearly 49% of them had zero to one year of birth spacing. Approximately 46% were educated up to the primary school level and 33.84% were illiterate. The majority of the study participants (76.62%) lived in joint families and more than half of them reported having six or more family members. The majority of them were holding "Below Poverty Line" cards (64.64%) and Antyodaya cards (26.84%). The demographic characteristics of study participants are presented in Table 2.

**Table 2:** Demographic characteristics of study participants (n=1,185) *Includes "do not wish to disclose" and "do not know" AN: antenatal; PN: postnatal; SD: standard deviation

Demographic characteristics	Total study participants, n (%)	AN women	PN women
		n (%)	n (%)
Age, years			
18–25	718 (60.59)	392 (62.92)	326 (58.01)
26–33	425 (35.86)	212 (34.03)	213 (37.90)
34–40	42 (3.54)	19 (3.05)	23 (4.09)
Mean ±SD	25.19 ±3.91	24.95 ±3.94	25.45 ±4.01
Age at marriage, years			
<20	258 (21.77)	16 (2.57)	242 (43.06)
20–25	681 (57.47)	376 (60.35)	305 (54.27)
>26	246 (20.76)	231 (37.08)	15 (2.67)
Mean ±SD	20.00 ±2.61	20.04 ±2.69	19.9 ±2.51
Age at first pregnancy, years			
<20	225 (18.99)	117 (18.78)	108 (19.22)
20–25	865 (73.00)	451 (72.39)	414 (73.67)
>26	95 (8.02)	55 (8.83)	40 (7.12)
Mean ±SD	21.67 ±2.76	21.67 ±2.71	21.70 ±2.81
Parity			
0	118 (9.96)	96 (15.41)	22 (3.91)
First	415 (35.02)	223 (35.79)	192 (34.16)
Second	390 (32.91)	183 (29.37)	207 (36.83)
Third or more	262 (22.11)	121 (19.42)	141 (25.09)
Gravida status			
First	408 (34.43)	222 (35.63)	186 (33.10)
Second	412 (34.77)	211 (33.87)	201 (35.77)
Third or more	365 (30.80)	190 (30.50)	175 (31.14)
Education			
Illiterate	401 (33.84)	223 (35.79)	178 (31.67)
Up to primary level	550 (46.41)	266 (42.70)	284 (50.53)
Secondary and higher education	234 (19.75)	134 (21.51)	100 (17.79)
Type of family			
Joint	908 (76.62)	481 (77.21)	427 (75.98)
Nuclear	277 (23.38)	142 (22.79)	135 (24.02)
Family size			
<3	51 (4.30)	24 (3.85)	27 (4.80)
4–5	464 (39.16)	272 (43.66)	192 (34.16)
6 or more	670 (56.54)	327 (52.49)	343 (61.03)
Religion			
Hindu	895 (75.53)	450 (72.23)	445 (79.18)
Muslim	278 (23.46)	166 (26.65)	112 (19.93)
Other	12 (1.01)	7 (1.12)	5 (0.89)
Socioeconomic status			
Below Poverty Line card holder	766 (64.64)	384 (61.64)	382 (67.97)
Antyodaya card holder	318 (26.84)	182 (29.21)	136 (24.20)
Above Poverty Line card holder	25 (2.11)	12 (1.93)	13 (2.31)
Other*	76 (6.41)	45 (7.22)	31 (5.52)

Availability of nutrition and healthcare services

The district is divided into four blocks: Dwarka, Kalyanpur, Khambhaliya, and Bhanvad, with a total population of 0.75 million as per the 2011 census. All four blocks had access to some kind of healthcare facility (government/private) for maternal healthcare. The District Hospital was situated in Khambhaliya block at a distance of 14-60 km from the other three blocks, and there was one sub-district hospital in Dwarka. Peripheral healthcare institutions were also functional in the district: 169 sub-centers, four Community Health Centers (CHCs), 23 Primary Health Centres (PHCs), and five Urban PHCs were present across four blocks, and one dispensary was functioning in Khambhaliya block. A total of 691 Anganwadi Centres (AWCs) were functioning across villages in the four blocks.

Utilization of antenatal and postnatal care services

Of the 1,185 AN and PN women, 180 (15.18%) did not avail any kind of ANC or PNC services. A detailed description of the service utilization is provided in Table 3. More than half of AN women (59.71%) and less than a quarter (16.85%) of PN women reported receiving iron-folic acid (IFA) tablets. Similarly, nearly half of the AN women (42.37%) and 13.32% of PN women reported receiving calcium tables. But the consumption of IFA tablets among AN women was 59.71% and that among PN women was as low as 18.68%, while the consumption of calcium tablets was 16.05 and 14.76% respectively.

**Table 3:** Nutritional and health services received by AN and PN women (n=1,185) AN: antenatal; PN: postnatal; IFA: iron-folic acid; Hb: hemoglobin; THR: take-home ration; ASHA: Accredited Social Activists; ANM: Auxiliary Nurse Midwife

Variables	AN women	PN women
	n=623	n=562
	n (%)	n (%)
Received any nutritional or health service		
Received	527 (84.59)	478 (76.73)
Not received	96 (15.40)	84 (13.48)
IFA supplement		
Received	372 (59.71)	105 (16.85)
Not received	252 (40.45)	457 (73.35)
Calcium supplement		
Received	264 (42.37)	83 (13.32)
Not received	359 (57.62)	479 (76.89)
Hb test		
Performed	480 (77.05)	464 (74.48)
Not performed	143 (22.95)	98 (15.73)
Counselling followed by Hb test		
Received	314 (50.40)	271 (43.49)
Not received	309 (49.59)	291 (46.70)
Anthropometry		
Availed anthropometry during Mamta Diwas	480 (77.05)	364 (58.43)
Not availed anthropometry during Mamta Diwas	143 (22.95)	198 (31.78)
THR		
Received	508 (81.54)	468 (75.12)
Not received	115 (18.46)	94 (15.09)
Double fortified test		
Received	461 (73.99)	363 (58.27)
Not received	162 (26.00)	199 (31.94)
Full immunization		
Received	527 (84.59)	478 (76.73)
Not received	96 (15.40)	84 (13.48)
Home visits by ASHA/ANM		
Up to two visits	303 (48.64)	281 (45.10)
Three-four visits	216 (34.67)	167 (26.81)
No visits	104 (16.69)	114 (18.29)

The majority of AN and PN women (77.05% and 74.48% respectively) underwent a Hb test either at the Anganwadi Center and Health and Wellness Center during Mamta Day or at a private service provider. But only half of them (50.40% and 43.49 respectively) received counseling after the Hb test. In general, a portion of AN and PN women reported receiving counseling at the Anganwadi Center. Uptake of other services such as anthropometry during Mamta Diwas (77.05% and 58.43% for AN and PN women respectively), take-home ration (THR) (81.54% and 75.12%), double fortified salt (73.99% and 58.27%), and full immunization (84.59% and 76.73%) were recorded. Nearly half (48.64%) of AN women and 45.10% of PN women reported up to two home visits in a month whereas four visits by Accredited Social Activists (ASHA) and/or Auxiliary Nurse Midwives (ANM) were reported by 17.17% of AN women and 13.35% of PN women. Institutional delivery was documented to be as high as 99%. But half of all the institutional deliveries had taken place in the private sector (51%). Access to health insurance schemes was restricted; only 18.4% of AN and PN women were enrolled in Mukhyamantri Amrutum (MA) Yojana while 17.7% had enrolled in Pradhan Mantri Matru Vandana Yojna.

As shown in Table 4, only 6.90% of AN women and 11.92% of PN women followed handwashing during all critical incidents. Most participants (87.9% of AN women and 87.37% of PN women) reported washing their hands with soap and about 77.85% of AN women and 74.38% of PN women used toilets for defecation.

**Table 4:** General hygiene among AN and PN women (n=1,185) AN: antenatal; PN: postnatal

Variables	AN women	PN women
	n (%)	n (%)
Handwashing		
In all critical incidents	48 (6.90)	67 (11.92)
Before preparing food	582 (93.42)	534 (95.02)
Before feeding the child	NA	505 (89.86)
After using the toilet	568 (91.17)	500 (88.97)
After handling cattle	193 (30.98)	228 (40.57)
After cleaning or mopping	520 (83.47)	435 (77.40)
After feeding the child	NA	380 (67.62)
Substance used for handwashing		
Handwash with soap	551 (88.44)	491 (87.37)
Handwash with plain water with/without ash	72 (11.56)	71 (12.63)
Household access to toilets		
Have a toilet at home	496 (79.61)	433 (77.05)
Usage of toilet	485 (77.85)	418 (74.38)

Anemia among AN and PN women

The prevalence of anemia among AN women (Hb: <11 g/dL) was 72.92%; the rates of mild (Hb: 10.0-10.9 g/dL), moderate (Hb: 7.0 to 9.9 g/dL), and severe (Hb: <7.0 g/dL) anemia among AN women were 38.96%, 33.91%, and 0.83% respectively. The prevalence of anemia among PN women (Hb: <12 g/dL) was 91.36%; the levels of mild (Hb: 10.0-11.9 g/dL), moderate (Hb: 8.0-10.9 g/dL), and severe (Hb: <8.0 g/dL) anemia among PN women were 26.13%, 63.93%, and 1.29% respectively. Hb levels among AN and PN women are presented in Tables 5-6.

**Table 5:** Anemia among pregnant women according to I-NIPI guidelines (n=480) I-NIPI: Intensified National Iron Plus Initiative

Hb levels	AN women
	n (%)
Severe anemia (<7.0 g/dL)	4 (0.83)
Moderate anemia (7.0–9.9 g/dL)	159 (33.13)
Mild anemia (10.0–10.9 g/dL)	187 (38.96)
Normal (>11 g/dL)	130 (27.08)

**Table 6:** Anemia among lactating women according to I-NIPI guidelines (n=463) I-NIPI: Intensified National Iron Plus Initiative

Hb levels	PN women
	n (%)
Severe anemia (<8.0 g/dL)	6 (1.29)
Moderate anemia (8.0–10.9 g/dL)	296 (63.93)
Mild anemia (11.0–11.9 g/dL)	121 (26.13)
Normal (>12 g/dL)	40 (8.64)

The present study found that age (p=0.045), birth spacing (p=0.014), and education (p=0.017) were significantly associated with anemia among AN women. An analysis of factors associated with anemia among AN and PN women are presented in Tables 7-8. Factors such as family type, family size, socioeconomic conditions, religion, availing nutritional and health services, and hygiene and sanitation practices were not significantly associated with anemia among pregnant women. For lactating women, parity (p=0.002), birth spacing (p=0.003), religion (p=0.041) and receiving THR (p=0.018) were significantly associated with anemia.

**Table 7:** Association of sociodemographic parameters with anemia among pregnant women (n=480) ^$^P-value is based on the chi-squared test of independence. ^#^Includes "do not wish to disclose" and "do not know". *Significant at 0.05 level SC/ST: Scheduled Caste/Scheduled Tribe; OBC: Other Backward Class; Hb: hemoglobin; IFA: iron-folic acid; THR: take-home ration

Parameters	Anemia (n=350)	Normal (n=130)	x^2 ^value	P-value^$^
	n (%)	n (%)		
Age, years				
18–25	226 (64.57)	77 (59.23)	6.198	0.0450*
26–33	116 (33.14)	44 (33.85)		
34–40	8 (2.29)	9 (6.92)		
Parity				
Zero	48 (13.71)	23 (17.69)	2.447	0.484
First	123 (35.14)	50 (38.46)		
Second	102 (29.14)	34 (26.15)		
Third or more	77 (22.00)	23 (17.69)		
Gravida status				
First	131 (37.43)	54 (41.54)	5.770	0.055
Second	102 (29.14)	47 (36.15)		
Third or more	117 (33.43)	29 (22.31)		
Birth spacing				
<1 year	142 (40.57)	61 (46.92)	10.490	0.014*
1 year	50 (14.29)	7 (5.38)		
2 years	52 (14.86)	13 (10.00)		
3 years or more	106 (30.29)	49 (37.69)		
Education				
Illiterate	122 (34.89)	41 (31.54)	8.119	0.017*
Up to primary level	160 (45.71)	48 (36.92)		
Secondary and higher education	68 (19.43)	41 (31.54)		
Type of family				
Joint	270 (77.14)	101 (77.69)	0.016	0.898
Nuclear	80 (22.86)	29 (22.31)		
Caste				
SC/ST	49 (14.00)	16 (12.31)	0.764	0.682
OBC	233 (66.57)	92 (70.77)		
General	68 (19.43)	22 (16.92)		
Religion				
Hindu	248 (70.86)	104 (80.00)	4.097	0.128
Muslim	99 (28.29)	25 (19.23)		
Other	3 (0.86)	1 (0.77)		
Socioeconomic status				
Below Poverty Line card holder	100 (28.57)	45 (34.62)	2.280	0.516
Antyodaya card holder	7 (2.00)	2 (1.54)		
Above Poverty Line card holder	215 (61.43)	76 (58.46)		
Other^#^	28 (8.00)	7 (5.38)		
Family members				
2–4	109 (31.14)	42 (32.31)	0.112	0.945
5–7	150 (42.86)	56 (43.08)		
8 or more	91 (26.00)	32 (24.62)		
Uptake of services				
Availed counseling before and/or after the Hb test	235 (67.14)	78 (60.00)	2.131	0.144
Not availed counseling	115 (32.86)	52 (40.00)		
Availed of IFA and calcium tablets	266 (76.00)	98 (75.38)	0.019	0.888
Not availed IFA and calcium tablets	84 (24.00)	32 (24.62)		
Availed THR	289 (82.57)	108 (83.08)	0.016	0.896
Not availed THR	61 (17.43)	22 (16.92)		
Availed anthropometry during Mamta Diwas	278 (79.43)	106 (81.54)	0.263	0.607
Not availed anthropometry during Mamta Diwas	72 (20.57)	24 (18.46)		
Hygiene and sanitation				
Handwashing in all critical incidents	23 (6.57)	4 (3.08)	2.18	0.139
No handwashing in all critical incidents	327 (93.43)	126 (96.92)		
Use of toilet for defecation	273 (78.00)	108 (83.08)	1.526	0.466
Not using toilets for defecation	6 (1.71)	2 (1.54)		
No response	71 (20.29)	20 (15.38)		

**Table 8:** Association of sociodemographic parameters with anemia among PN women (n=463) ^$^P-value is based on the chi-squared test of independence. ^#^Includes "do not wish to disclose" and "do not know". *Significant at 0.05 level SC/ST: Scheduled Caste/Scheduled Tribe; OBC: Other Backward Class; Hb: hemoglobin; IFA: iron-folic acid; THR: take-home ration

Parameters	Anemia (n=423)	Normal (n=40)	x^2 ^value	P-value^$^
	n (%)	n (%)		
Age, years				
18–25	235 (55.56)	27 (67.50)	2.160	0.339
26–33	170 (40.19)	12 (30.00)		
34–45	18 (4.26)	1 (2.50)		
Parity				
Zero	16 (3.78)	1 (2.50)	14.165	0.002*
First	133 (31.44)	23 (57.50)		
Second	159 (37.59)	11 (27.50)		
Third or more	115 (27.19)	5 (12.50)		
Gravida status				
First	143 (33.81)	20 (50.00)	4.839	0.088
Second	153 (36.17)	13 (32.50)		
Third or more	127 (30.02)	7 (17.50)		
Birth spacing				
0	134 (31.68)	24 (60.00)	13.582	0.003*
1 year	51 (12.06)	3 (7.50)		
2 years	80 (18.91)	6 (15.00)		
3 or more years	158 (37.35)	7 (17.50)		
Education				
Illiterate	135 (31.91)	9 (22.50)	2.809	0.245
Up to primary level	212 (50.12)	20 (50.00)		
Secondary and higher education	76 (17.97)	11 (27.50)		
Type of family				
Joint	327 (77.30)	31 (77.50)	0.000	0.977
Nuclear	96 (22.70)	9 (22.50)		
Caste				
SC/ST	68 (16.08)	5 (12.50)	1.218	0.748
OBC	260 (61.47)	28 (70.00)		
General	73 (17.26)	5 (12.50)		
Other^#^	2 (5.00)	2 (5.00)		
Religion				
Hindu	336 (79.43)	31 (77.50)	6.35	0.041*
Muslim	84 (19.86)	7 (17.50)		
Other^#^	336 (79.43)	31 (77.50)		
Socioeconomic status				
Below Poverty Line card holder	101 (23.88)	13 (32.50)	1.530	0.675
Antyodaya card holder	9 (2.13)	1 (2.50)		
Above Poverty Line card holder	289 (68.32)	24 (60.00)		
Other^#^	101 (23.88)	13 (32.50)		
Family members				
2–4	72 (17.02)	7 (17.50)	0.975	0.613
5–7	222 (52.48)	18 (45.00)		
8 or more	129 (30.50)	15 (37.50)		
Uptake of services				
Availed counseling after Hb test	248 (58.63)	18 (45.00)	2.776	0.095
Not availed counseling	175 (41.84)	22 (55.00)		
Availed of IFA and calcium tablets	309 (73.05)	36 (90.00)	2.122	0.145
Not availed IFA and calcium tablets	114 (26.95)	4 (10.00)		
Availed THR	272 (64.30)	31 (77.50)	5.528	0.018*
Not availed THR	151 (35.70)	9 (22.50)		
Availed anthropometry during Mamta Diwas	72 (17.02)	7 (17.50)	2.814	0.093
Not availed anthropometry during Mamta Diwas	222 (52.48)	18 (45.00)		
Hygiene and sanitation				
Handwashing in all critical incidents	53 (12.53)	2 (5.00)	1.979	0.159
No handwashing in all critical incidents	370 (87.47)	38 (95.00)		
Use of toilet for defecation	318 (75.18)	29 (72.50)	1.304	0.520
Not using toilets for defecation	9 (2.13)	2 (5.00)		
No response	96 (22.70)	9 (22.50)		

## Discussion

This survey from the Devbhumi Dwarka district highlights the prevalence of anemia, utilization of services, and factors associated with anemia among AN and PN women. A high prevalence of anemia was observed among AN and PN women. A study conducted by Kalaivani showed that over 70% of pregnant women in the country were anemic [33]. In the present study, a slightly higher prevalence rate of anemia among AN women (72.92%) (Hb: <11 g/dL) was observed; the levels of moderate (Hb: 7-9.9 g/dL) and severe (Hb: <7 g/dL) anemia were 33.91% and 0.83% respectively. Conversely, a very high prevalence (82.9%) was observed by Viveki et al. in 2012, and an 81.95% prevalence was reported by Singh et al. [34,35]. Two other Indian studies have reported a prevalence of anemia of 62.3% and 64% respectively [36-37]. However, a lower prevalence than the current study was reported in NFHS-4 (49.7%) [3].

In the present study, the overall prevalence of anemia among PN women was 91.36% and that of moderate and severe anemia was 63.93% and 1.29% respectively. Siddiqui et al. [37] reported a 63% prevalence of anemia among PN women. Other recent studies in different regions of the world, such as Myanmar (60.3%), China (32.7%), and Ethiopia (28.7%), reported a prevalence lower than in the current study [38-41].

A major proportion of anemia prevalence involved mild and moderate levels of anemia. According to WHO [29-31] and NIPI guidelines [32], mild and moderate anemia can be treated with the consumption of IFA tablets. Thus, counseling patients on consuming iron-rich food and IFA supplementation after the Hb test has been recommended by both WHO and NIPI guidelines. However, nearly half of the women (49%; 59% of AN women, and 46.70% of PN women) did not receive counseling followed by a Hb test. Furthermore, intake of supplements was poor among AN women (59.71%) and PN women (18.68%). The consumption trend was also poor for calcium tablets: 16.05% among AN women and 14.76% among PN women. Similar findings were reported in another study [42]. A study by Ghosh-Jerath et al. revealed that only a fourth of the study population reported receiving counseling [42]. Such limited availability of counseling after the Hb test could limit the gains expected out of these sessions in bringing about behavioral change with respect to the consumption of IFA and calcium tablets that are most critical in pregnancy and post-pregnancy. Ghosh-Jerath et al. [42] have explored the effectiveness of utilizing frontline workers, especially ASHAs in decentralizing the workload of health promotion to effectively diffuse counseling services between health facilities and community health workers. However, further research on this aspect in the rural Indian context is required to demonstrate the effectiveness of the role of ASHAs in nutrition counseling and improving nutrition and health outcomes.

Utilization of nutritional and health services was sub-optimal, especially PNC. Though most of the women surveyed received some kind of ANC or PNC, complete ANC and PNC were limited. In our study population, 15.18% of women did not avail of any ANC or PNC services. This is in line with the findings of Ghosh-Jerath et al. in Delhi [42], Singh et al. Uttar Pradesh [43], and the NFHS-4 data [3], but lower when compared to a cross-sectional survey conducted by Kardalkar and Sherkhane in an urban slum of Gujarat [24].

ANC and PNC are the key services that AN and PN women should receive as part of a broad range of health promotion and prevention services. The WHO [29,30] recommends a minimum of four ANC visits, ideally at 16, 24-28, 32, and 36 weeks, and a minimum of four PNC visits, ideally within 6-12 hours after birth, and follow-up visits from three to six days, at six weeks, and then at six months. Additional three to four PNC visits are recommended in the case of LBW babies: on days 14, 21, and 28 as per the Integrated Management of Neonatal and Childhood Illness (IMNCI) guidelines [44]. But the present study revealed very few home visits; only 15.36% of AN and PN women (17.17% among AN women; 13.35% of PN women) reported four home visits by ASHA/ANM, which is lower than the 51.2% reported in the NFHS-4 [3] and very low when compared to increase in the rate of institutional delivery in Devbhumi Dwarka, which was 99%, higher than the national rate of institutional delivery (79%) [3].

Home visits are considered effective in improving maternal and child health outcomes [44,45]. Systematic reviews of studies conducted in South Asian and African countries have conclusively shown that home visits during AN and PN periods can improve both the demand for and use of AN, intrapartum, and PN care services, and potentially reduce maternal and newborn mortality by at least 15-20% [46,47].

Studies on the impact of AN dietary advice, nutrition education, and counseling with or without nutrition supplementation have reported improved dietary intake and weight gain in mothers, reduced risk of anemia and preterm delivery, and increased head birth weight [48,49]. In the present study, handwashing practices in all critical incidents and consumption of IFA and calcium tablets were sub-optimal, underscoring the importance of nutrition education and counseling. ANC/PNC services are considered opportunities for the dissemination of information, education, and communication to promote behavioral changes [50]. This gap can be bridged by nutrition-related training of frontline health workers, which can play an important role in improving care providers’ health and nutrition-related practices [51,52].

Various sociodemographic factors influence maternal anemia. A study conducted in Madhya Pradesh in 2014 [53] identified that a number of demographic and socioeconomic factors such as age, education, religion, family type, and family size were significantly associated with anemia among AN women. In the present study, age, birth spacing, and education were associated with anemia, which endorses the findings of a few Indian studies [54,55].

Studies have revealed that factors such as maternal age, educational status of the mother, parity, ANC visits, history of terminated pregnancies, health insurance, maternal occupation, religion, marital status, toilet facility, place of delivery, iron supplementation during pregnancy, parity, birth spacing, gravida, and region were associated with anemia among PN women [36,37,56,57]. However, the current study showed that factors such as parity (p=0.002), birth spacing (p=0.003), religion (p=0.041), and receipt of THR (p=0.018) are significantly associated with anemia among PN women.

The study's findings can be utilized for planning effective interventions to reduce maternal anemia and improve the utilization of nutrition and healthcare services. In summary, we recommend (i) training of frontline health workers in nutrition education and counseling, (ii) counseling of AN and PN women, especially after the Hb test, (iii) strengthening coverage of ANC and PNC services, including the provision of IFA, calcium and food supplements, (iv) timely treatment or referral of PLW with severe anemia, and (v) promoting handwashing practices. Future studies may identify and address barriers to the utilization of AN and PN services, food consumption patterns among AN and PN women, and social norms and cultural practices that adversely influence maternal and childcare practices.

Strengths and limitations

The major strength of the current study is its triple study setting, which is generally overlooked. Nevertheless, it is important to note that the Hb level was considered based on the last Hb test recorded in the Mamta Card (Mother and Child Protection Card); hence there may be a few errors in reporting Hb test results. However, the statistically calculated sample size was sufficient to make up for any shortcomings in the findings. While I-NIPI guidelines of the Government of India recommend a digital haemoglobinometer, Hb estimation in the district is still done by conventional Sahli’s method, which has its own limitations and may introduce serious bias in estimation. Validation through a digital haemoglobinometer and estimation of serum ferritin can guide better program management strategies.

## Conclusions

The high prevalence of anemia among AN and PN women with sub-optimal utilization of nutritional and healthcare services in Devbhumi Dwarka is a concern. The study clearly highlights the urgent need to bridge the gaps in ANC and PNC services and advocates for comprehensive nutrition education and counseling services for better maternal and child health outcomes.
